# Postoperative intussusception: a rare but critical complication in adult patients with Crohn’s disease – case report and literature review

**DOI:** 10.1515/iss-2023-0012

**Published:** 2023-10-06

**Authors:** Sophie M. Eschlböck, Benjamin Weixler, Carl Weidinger, Ioannis Pozios

**Affiliations:** Department of General and Visceral Surgery, Charité – Universitätsmedizin Berlin, corporate member of Freie Universität Berlin and Humboldt-Universität zu Berlin, Charité – Campus Benjamin Franklin, Hindenburgdamm 30, 12203 Berlin, Germany; Department of Gastroenterology, Rheumatology and Infectiology, Charité – Universitätsmedizin Berlin, corporate member of Freie Universität Berlin and Humboldt-Universität zu Berlin, Hindenburgdamm 30, 12203 Berlin, Germany

**Keywords:** Crohn’s disease, intussusception, postoperative complication

## Abstract

**Objectives:**

Postoperative entero-enteric intussusception is a rare complication in adult patients with Crohn’s disease (CD). The knowledge of this distinct complication and its timely diagnosis and therapy are of utmost importance to prevent fatal intestinal necrosis. There is no consensus about the optimal management of postoperative entero–enteric intussusception, although surgical exploration is widely advised.

**Case presentation:**

In this report we describe an unusual case of postoperative jejuno–jejunal intussusception following small bowel resection in a patient with stricturing CD. Furthermore, this report offers an overview of the available literature and summarizes the best approach and management strategies for adult intussusception associated with CD.

**Conclusions:**

Delay in diagnosis and therapy can lead to life-threatening complications. Early diagnosis and emergent surgical treatment prevent intestinal necrosis and reduce the risk of short bowel syndrome.

## Introduction

Crohn’s disease (CD) is a chronic inflammatory disease of the gastrointestinal tract, assumed to result from genetic and environmental factors as well as altered gut microbiota, leading to dysregulated immune responses [1]. It is associated with various complications such as strictures, fistulas and abscesses, often requiring surgery [2], [3], [4].

Intussusception is defined as the invagination of a proximal segment of the gastrointestinal tract, called intussusceptum, into the lumen of an adjacent one, called intussuscipiens. It can lead to impaired peristalsis and intestinal obstruction and bears the risk of ischemia, perforation, and peritonitis [5, 6]. While intussusception is the main cause of intestinal obstruction in children, it represents only 1–5 % of small bowel obstructions in adults [5, 7, 8].

So far, cases describing intussusception as a postoperative complication in patients with CD have been rarely reported in the literature [9], [10], [11], [12], [13], [14]. Here, we present a case of jejuno–jejunal intussusception in a postoperative setting following small bowel resection and offer an overview of the literature reporting adult intussusception associated with CD.

## Case presentation

**Figure 1:**
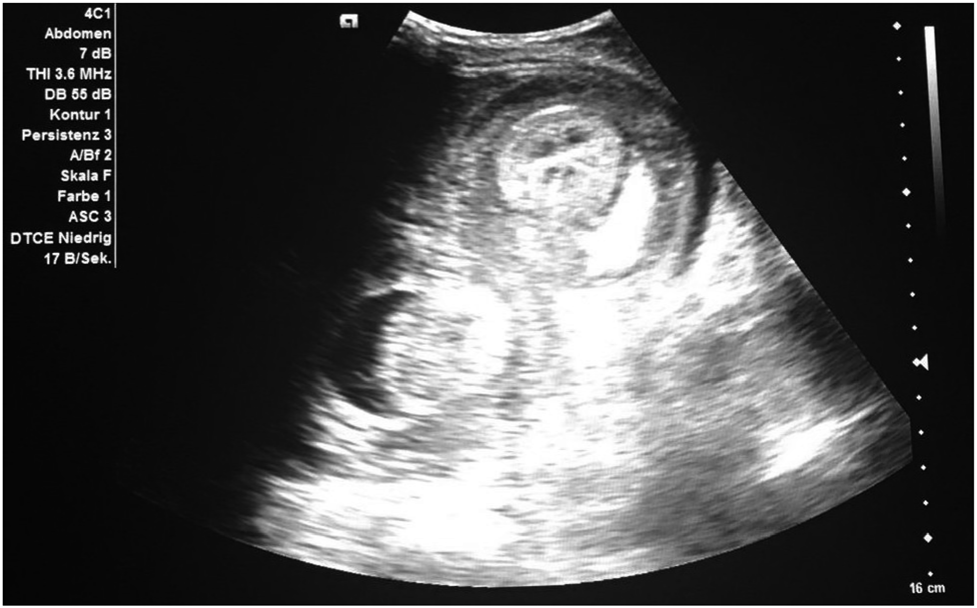
Bedside abdominal ultrasound scan revealed “target sign”.

**Figure 2:**
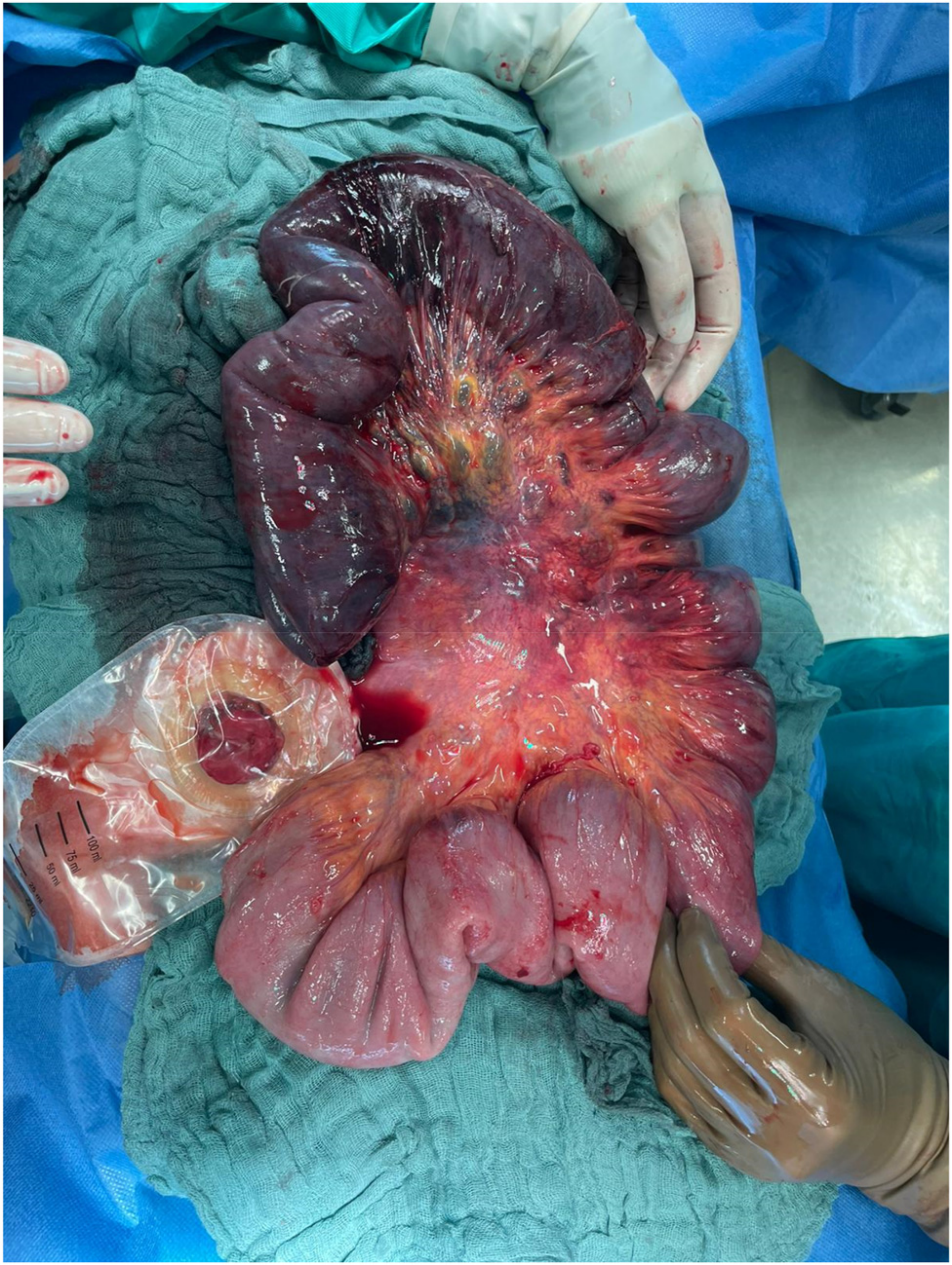
Extent of ischemic small bowel after disinvagination.

A 27-year-old male patient with CD was transferred to our tertiary referral center with acute mechanical ileus due to fibrostenosis in the ileum. The patient, who was cachectic on presentation, had been first diagnosed with CD at the age of 21 and had been under treatment with Azathioprine for many years. In the past, whenever he presented with symptoms referred to ileal stenosis, the therapy was temporarily supplemented with Prednisone. Follow-up examinations or colonoscopies were not offered to the patient before. Magnetic resonance tomography showed fibrostenosis in the ileum with pre-stenotic dilatation (8 cm in diameter) of the small bowel. We performed a laparoscopic ileal segment resection with side-to-side ileo–ileal isoperistaltic (Kono-S) anastomosis and protective ileostomy. Protective ileostomy was created because of the reduced nutritional status and prior therapy with high dose prednisone before transfer. On postoperative day two the patient presented with severe bleeding from the ileostomy, excessive vomiting, and abdominal pain. He was tachycardic and hypotonic and repeated blood gas analysis showed a decrease in hemoglobin. The clinical examination revealed a palpable periumbilical tender mass. Abdominal ultrasound showed an intussusception with the typical “target sign” (Figure 1) [6, 15, 16]. Based on the severe symptoms and imaging findings the patient was immediately taken to the operating room. An emergency laparotomy revealed an already ischemic jejuno–jejunal intussusception, beginning from the ligament of Treitz, approximately 80 cm in length (Figure 2). Manual reduction was performed and bowel perfusion almost entirely recovered, except for a bowel segment 20 cm distal of the ligament of Treitz. This segment was necrotic over a length of 30 cm and had to be resected. Due to the unstable general condition of the patient, we decided for a damage control concept and left blind bowel ends after resection of the necrotic loop, so that the patient could be stabilized in the intensive care unit. After 24 h, an end jejunostomy was then created. One week after intussusception, continuity could be restored with side-to-side jejuno–jejunostomy. The further postoperative recovery was uneventful and the patient could be discharged and received regular follow-up examinations in our outpatient clinic. After seven months, ileostomy reversal surgery was performed without any complications. Postoperatively the patient was put on anti-TNF-alpha therapy as medical prophylaxis and remains asymptomatic with no recurrence six months after ileostomy reversal.

## Discussion

Intussusception is primarily a pediatric disease but can also occur on rare occasion in adults [8]. Compared to children, the condition in adults differs concerning the pathological lesion and therapeutic options [6]. In children, intussusception is usually idiopathic and benign and can sufficiently be treated with pneumatic or hydrostatic reduction in most cases [5, 6, 17]. Adult intussusception represents only 5 % of all intussusception cases and a pathological lesion as a lead point is usually found, bearing a significant risk for malignancy [5, 6, 8, 15, 18].

Intussusception in patients with CD is very uncommon and therefore represents a challenging clinical scenario [6, 12]. Due to the rarity of this complication and the potentially fatal consequences we performed a comprehensive review of the existing literature and identified 16 reported cases of adult intussusception (minimum age of 16) associated with CD worldwide from 1979 to the present day [9], [10], [11], [12], [13], [14, 17], [18], [19], [20], [21], [22].

### Clinical presentation and diagnosis

In the presented case the patient had a long-standing intestinal dilatation due to the chronic obstructive Crohn’s lesion. We assume that the intussusception occurred after resection of the stenosis when proper peristalsis resumed.

He presented with abdominal pain, intestinal bleeding, and an abdominal tender mass. Also, in children the clinical presentation is a triad of abdominal pain, bloody diarrhea, and a palpable mass. However, adults do not necessarily present with this typical triad [5].

In the literature symptoms ranged from light intermittent abdominal pain over a longer period of time to acute severe crampy abdominal pain, vomiting and intestinal bleeding [9], [10], [11], [12], [13], [14, 19], [20], [21], [22]. In eleven of the cases, intussusception developed postoperatively in patients with CD who had undergone surgery for complications related to the disease. The time between the operations and occurrence of the intussusception ranged from three days to several years [9], [10], [11], [12], [13], [14].

In five cases intussusception was reported without prior surgery [17, 19], [20], [21], [22]. The transient occurrence of intussusception in patients with CD might be explained by a segment of thickened, inflamed bowel wall acting as a lead point or the interruption of peristalsis through submucosal bowel edema or a bowel wall lesion such as an abscess or a pseudopolyp [9, 11, 13, 14, 17, 19]. Fernandez et al. report that some cases of small bowel intussusception have not only been observed in patients with CD but also in adult coeliac disease and other benign conditions and could be the first manifestation of these diseases [20]. Due to the bowel changes caused by CD, other authors argue that intussusception might present as a transient phenomenon in affected patients and spontaneous resolution can occur with conservative management [11, 13, 19].

As intussusception in adults presents considerable variability in the patient’s clinical presentation, it is recommended to use radiological examinations such as abdominal X-ray, barium enema, abdominal ultrasound, or abdominal computed tomography (CT) to make a correct diagnosis [23]. In the presented case ultrasound revealed the intussusception by showing the typical “target sign” in the transverse scan. However, ultrasound may not always be able to detect intussusception. The widespread use of CT has significantly increased the detection of adult intussusception and contrast enhanced CT scan is considered the gold standard for intussusception diagnosis [6, 23, 24], while X-ray and barium enema are obsolete for this emergency situation.

### Therapy

Optimal treatment is still unclear as no consensus exists about the indications of intussusception reduction or the necessity of emergent primary resection [6, 18, 20]. Because of a significant risk for malignancy in adults, preoperative radiologic decompression is not recommended in adults in contrast to children [5, 6, 8, 15, 18].

Concerning small bowel intussusception, resection may not be necessary in all cases since the underlying condition might be benign [6, 18]. Furthermore, in patients with CD the preservation of bowel length is imperative to avoid short bowel syndrome in the further course of the disease. However, the damage caused by the intussusception by the time the patient is taken to the operating room might not allow manual reduction without resection of the impaired intestinal segment [12, 20]. In the reported case, a manual reduction of the intussusception was possible, but not all of the intussusceptum could be preserved due to the irreducible necrosis of the intestine. As our patient finally underwent a resection of only 30 cm, he has not demonstrated any nutritional disorder and gained considerable weight since ileostomy reversal.

As colonic intussusception in adults shows a high rate of malignancies, some authors argue that primary resection without attempting reduction represents the optimal management to avoid possible tumor dissemination [6, 17, 20]. Draganic et al. suggest that after resection of the colonic intussusception, a safe anastomosis may not be possible if the colon is affected by active CD, and total colectomy may be preferred to segmental colectomy [17].

In the literature reviewed, five cases required laparotomy and the intussusception could be reduced (Table 1) [9, 10, 12]. Six patients required laparotomy and bowel resection [10], [11], [12, 14, 20], while three did not require surgery and were reducible by conservative treatment, such as nil per oral, intravenous fluids, parenteral nutrition, and antibiotics [13, 19, 21, 22]. Three of the six patients that required bowel resection were initially treated conservatively but had to undergo surgery after three or seven days or one month, respectively, due to no improvement or aggravation of their condition [10, 12, 20]. Delayed laparotomy showed necrosis or perforation of the bowel and up to 150 cm of the small intestine was lost, leaving the patient at risk for short bowel syndrome [12]. None of the publications mentioned above report malignancy as the cause for intussusception. In the presented case no Crohn’s lesion or sign of malignancy were found in the resected specimen.

**Table 1:** Literature overview: intussusception in adult patients with Crohn’s disease.

Author	Symptoms	Surgery prior to intussusception	Time between surgery and intussusception	Location of intussusception	Therapy	Course
Greenstein et al. [9]						
Case 1	NA	Ileocecal resection, ileoascending colostomy, anterior resection with coloproctostomy, loop ileostomy	1 month	Jejuno–jejunal	Laparotomy, manual reduction	Asymptomatic 2 years postoperatively
Case 2	Severe, crampy left upper quadrant pain, visible peristalsis in the upper abdomen	Ileocecal resection, ileoascending colostomy	6 weeks	Jejuno–jejunal	Laparotomy, manual reduction	Asymptomatic 2 years postoperatively
Hertz et al. [10]						
Case 1	NA	Ileocecal resection, ileoascending anastomosis, anterior resection of the sigmoid and proximal rectum, loop ileostomy	1 month	Jejuno–jejunal	Laparotomy, manual reduction	Asymptomatic 5 years postoperatively
Case 2	Severe left upper quadrant pain, visible upper abdominal peristalsis	Ileocecal resection, ileoascending anastomosis	6 weeks	Jejuno–jejunal	Laparotomy, manual reduction	Asymptomatic 3 years postoperatively
Case 3/1 (same patient)	Left abdominal mass, vomiting	Resection of distal ileum, ileoascending anastomosis	Same hospital stay	Jejuno–jejunal	Conservative (patient became clinically well without treatment)	
Case 3/2 (same patient)			1 month	Jejuno–jejunal	Resection of necrotic jejunum due to intestinal perforation	Asymptomatic 1 year postoperatively
Knowles et al. [11]						
Case 1	Intermittent abdominal pain for 2 months	3 laparotomies with bowel resections	NA	Small bowel	Laparotomy with resection of distal ileum	NA
Case 2	Severe, crampy abdominal pain for 4 days, nausea	Resection of ileum after perforation	Several years	Ileum	Laparotomy with small bowel resection	NA
Catalano et al. [21]	Abdominal pain for 3 months, palpable abdominal mass	None	-	Colo–colic	None	Enema 1 year later still showed chronic intussusception
Shah et al. [22]	Abdominal pain, nausea, bilious vomiting	None	-	Entero–enteric	Conservative	Discharged after 17 days
Atten et al. [14]	Intermittent, colicky midabdominal pain, bloody diarrhea	Segmental resection of left colon	8 years	Colo–colic	Laparotomy, right hemicolectomy with end loop ileostomy	NA
Kihiczak et al. [19]	Severe intermittent abdominal pain	None	-	Ileo–colic	Conservative	Discharged after 8 days
Dragani et al. [17]	Severe colicky lower abdominal pain, minor rectal bleeding, abdominal distension for 3 days	None	-	Colo–colic	Barium enema	Asymptomatic at 6 months after discharge
Fernández et al. [20]	Central abdominal pain, vomiting, and bloody stool for previous 2 days	None	-	Distal ileum	Initially conservative, laparotomy after 3 days with reduction and bowel resection	Asymptomatic at 6 months follow up
Uchino et al. [12]						
Case 1	Abdominal pain, nausea, upper left quadrant abdominal distension	Ileocecal resection, ileoascending colostomy	3 days	Jejuno–jejunal	Initially conservative; laparotomy and resection of incarcerated jejunal intussuscipiens on the 7th postoperative day	Asymptomatic 1 year postoperatively
Case 2	Abdominal pain, nausea, upper left quadrant abdominal distension	Ileocecal resection	14 days	Small intestine	Laparotomy, manual reduction	Discharged on 27th postoperative day
Pandit et al. [13]	Colicky, intermittent pain in the left hypochondrium	Stoma closure after jejunal and ileal resection, double-barrel jejunostomy	13 days	Jejuno–jejunal	Conservative	Discharged on 20th postoperative day

## Conclusions

Postoperative intussusception is a very rare complication in adult patients with CD. The patients’ clinical presentation can often be nonspecific and make the diagnosis difficult. However, physicians should be familiar with this differential diagnosis since delay in diagnosis and therapy can lead to life-threatening complications. Intussusception due to malignancy plays a marginal role in patients with CD. Early diagnosis and emergent surgical treatment prevent intestinal necrosis and reduce the risk of short bowel syndrome. Surgical management should be individually evaluated based on the intraoperative findings, but a stepped approach should be preferred in patients with CD to minimize the resected bowel region.

## Supplementary Material

Supplementary Material
