# Supplementary material for: Postoperative intussusception: a rare but critical complication in adult patients with Crohn’s disease – case report and literature review
Source: Innov Surg Sci. 2023 Oct 6;8(1):3–8. doi: 10.1515/iss-2023-0012 (PMC10576550; doi:10.1515/iss-2023-0012)
Supplement: Supplementary file 1 — Supplementary Material [file j_iss-2023-0012_suppl_001.pdf]

## Reviewer Assessment

# S. M. Eschlböck et al.: Postoperative intussusception: a rare but critical complication in adult patients with Crohn’s disease - Case report and literature review

### Reviewers’ Comments to Original Submission

**Reviewer 1: Schiedeck, Thomas**

Date received: 24-May-2023
Reviewer recommendation: **Accept in present form**
Reviewer overall scoring: **Medium**

**Comments to author:**
The presented case-report is a very good example for these rare postoperative complication. Handling of the situation and treatment was very consise and is appropriate to spread the knowledge about invagination and intussusception. I would suggest to publish the contribution in the presented form without any further correction.

**Reviewer 2: anonymous**

Date received: 10-Jul-2023
Reviewer recommendation: **Accept in present form**
Reviewer overall scoring: **Medium**

**Comments to author:**
Nice presentation of a rare, but significant complication in surgery for Crohn's disease. The readers of the article get relevant information about this complication and may consider it when treating patients with a somewhat complicated postoperative course after surgery for Crohn's disease.
I recommend a publication in present form.
